# A stratified survey dataset on internship experience, competencies, psychological capital, and employability of vocational students in Aceh, Indonesia

**DOI:** 10.1016/j.dib.2026.112992

**Published:** 2026-06-20

**Authors:** Didi Pianda, Hilmiana Hilmiana, Sunu Widianto, Dina Sartika

**Affiliations:** Faculty of Business, Economics and Management, Padjadjaran University, Bandung, Indonesia

**Keywords:** Vocational students, Internship experience, Competence, Psychological capital, Employability, Aceh Indonesia

## Abstract

This article presents a survey dataset regarding internship experience, competence, psychological capital, and employability among vocational students in Aceh Province, Indonesia. The data were collected from 10 September to 30 December 2022 using a quantitative survey approach. The sample consists of 266 vocational students who completed internship programs in private and public industries. To ensure rigor, respondents were selected using a proportional stratified random sampling technique, with the minimum sample size determined via the Isaac and Michael [1] formula based on a total provincial population of 15,435 students. Demographic variables captured include age, gender, school status, and accreditation. This dataset allows researchers to evaluate the relationship between soft skills (Psychological Capital) and hard skills (Competence) in forming graduate employability, providing a valuable baseline for educational policy evaluation in developing regions with unique geopolitical contexts.

Specifications Table

**Table:** 

Subject	Education and Management
Specific subject area	Organizational behavior, organizational psychology, human resource management, and education
Type of data	Descriptive data and raw.
Data collection	Proportional Stratified Random Sampling was used. The sample size was calculated using the Isaac and Michael [1] formula based on a total population of 7373 vocational students (Grade XII) across 7 districts in Aceh Province. The data were collected through questionnaires, for students’ internship experience adopted by [[2], [3]], competencies [4], psychological capital [[5], [6]], and employability [[7], [8]]. In this context, respondents communicated some information related to the survey, filling in and collecting data manually using paper and pen. At the end of the session, the survey was completed and submitted immediately. Respondents were guided, and clear instructions were given to prevent misunderstanding of the answers.
Data source location	Institution: Vocational school City/Town/Region: Banda Aceh, Provinsi Aceh Country: Indonesia
Data accessibility	Repository name: Mendeley Data Data identification number: 10.17632/hmn3b4c6c4.4 Direct URL to data: https://data.mendeley.com/datasets/hmn3b4c6c4/4 See Reference [9]
Related research article	Pianda, D., Hilmiana, Widianto, S., & Sartika, D. (2025). The influence employability of vocational students through internship experiences and 21st-century competencies: a moderated mediation model. *Cogent Education, 12*(1). https://doi.org/10.1080/2331186X.2025.2476285. See Reference [10] Pianda, D., Hilmiana, H., Widianto, S., & Sartika, D. (2024). The impact of internship experience on the employability of vocational students: a bibliometric and systematic review. *Cogent Business & Management*, 11(1). https://doi.org/10.1080/23311975.2024.2386465. See Reference [11] Pianda D, Agustina LF, Indiyati D, Hilmiana, Widianto S, Sartika D, Darmawan J (2026;), “Fostering green employability: a systematic review and bibliometric analysis using biblioshiny of vocational education’s role in the sustainable transition”. *Higher Education, Skills and Work-based Learning*, Vol. ahead-of-print No ahead-of-print. https://doi.org/10.1108/HESWBL-01-2026-0006. See Reference [12]

## 1 Value of the Data

• This is the first open-access dataset capturing the psychological capital and employability of vocational students in Aceh Province, a region with a unique post-conflict history and the implementation of Sharia Law, offering a distinct cultural variable for comparative global studies.
• The dataset provides granular item-level responses (Likert scale), allowing other researchers to test the validity of Western-derived scales (e.g., PsyCap) when applied to vocational students in developing regions.
• The data offers empirical evidence for policymakers to evaluate the effectiveness of the industrial internship program (Prakerin) in bridging the gap between vocational education and industry needs.
• The raw data serves as a training set for researchers developing predictive models on how soft skills (Psychological Capital) mediate the relationship between hard skills (Competence) and graduate employability.

## 2 Background

Student employability in vocational education has become a critical global concern, particularly in aligning graduate skills with dynamic labor market demands [[10], [13]]. However, there is a significant scarcity of empirical data regarding vocational students in Aceh Province, a region characterized by a unique geopolitical context following a prolonged conflict and the implementation of Sharia Law. This distinct cultural and educational environment may influence students' psychological capital and internship experiences differently compared to other regions in Indonesia or globally [[14], [15]].

This data collection was driven by the findings of our recent bibliometric and systematic review [11], which highlighted that existing literature largely focuses on direct competence measurements, often overlooking the mediating role of Psychological Capital (PsyCap), consisting of hope, efficacy, resilience, and optimism in forming work readiness [11,12]. Furthermore, while internships (*Prakerin*) are mandatory in Indonesian vocational schools, data linking specific internship quality metrics to employability outcomes remains fragmented [[16], [17], [18]]. The urgency of such data collection is further supported by recent studies indicating that accurate identification of youth characteristics within Vocational Education and Training (VET) systems is pivotal for designing targeted strategies to improve employability [19]. Similarly, recent data initiatives in other complex geopolitical contexts such as the intersectional analysis of living conditions in Tumaco, Colombia [20], demonstrate the critical value of localized datasets in understanding workforce dynamics in the Global South.

This dataset was collected to bridge these gaps by providing a comprehensive baseline on how internship experiences and psychological attributes interact to form employability in a post-conflict, developing region. The data support the “Triple-Helix” logic (government-school-industry) by offering granular evidence to evaluate whether current vocational curricula in Aceh successfully foster the soft skills required by modern industry [14,[21], [22], [23]].

## 3 Data Description

**Fig. 1:**
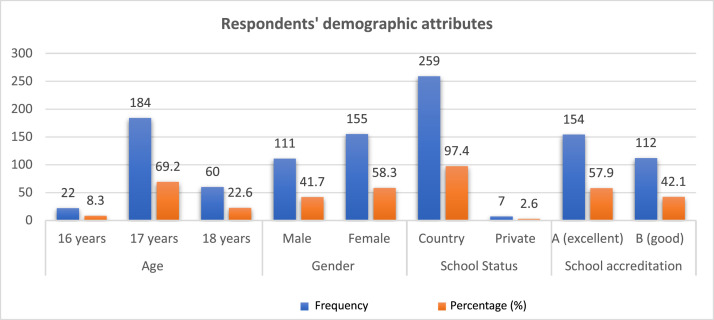
Respondents' demographic characteristics.

The data on demographic characteristics were provided, including age, gender, school status, and school accreditation. The observed frequency data of various demographic characteristics for age, where 16, 17, and 18-year-olds were observed in 22 (8.3%), 184 (69.2%), and 60 (22.6%) respondents, respectively. Concerning the gender characteristics, 111 (41.7%) and 156 (58.3%) respondents were reported to be males and females, respectively. Furthermore, 259 (97.4%) and 7 (2.6%) respondents were observed in private and public schools, respectively. In this context of accreditation, where A (excellent) and B (good) accredited schools were observed in 154 (57.9%) and 112 (42.1%) respondents, as presented in Table 1 and Fig. 1. In addition to the demographic profile, the statistical properties of the research variables were evaluated. The variables' descriptive statistics, including means, standard deviations, minimum, and maximum values, are detailed in Table 2.

**Table 1:** Respondents' demographic attributes.

Demografic Characteristics	Item	Frequency	Percentage (%)
Age	16 years	22	8.3
	17 years	184	69.2
	18 years	60	22.6
**Total**		**266**	**100**
Gender	Male	111	41.7
	Female	155	58.3
**Total**		**266**	**100**
School Status	Country	259	97.4
	Private	7	2.6
**Total**		**266**	**100**
School Accreditation	A (excellent)	154	57.9
	B (good)	112	42.1
**Total**		**266**	**100**

**Table 2:** Variables' descriptive statistics.

Variable	N	Min	Max	Mean		Std. Deviation	Skewness		Kurtosis	
				Statistic	Std. Error		Statistic	Std. Error	Statistic	Std. Error
Students’ internship experience	266	1.00	5.00	4.008	0.402	0.656	−1.059	0.149	3.649	0.298
Competence	266	1.00	5.00	3.880	0.388	0.633	−0.798	0.149	2.237	0.298
Psychological capital	266	1.00	5.00	4.124	0.407	0.664	−1.389	0.149	5.440	0.298
Employability of Vocational Students	266	1.00	5.00	4.000	0.402	0.656	−0.889	0.149	2.597	0.298

To evaluate the normality of the dataset, descriptive statistics, including Skewness and Kurtosis, were analyzed. The analysis reveals that the Skewness values range from −2.150 to −0.944, indicating a negatively skewed distribution. The Kurtosis values range from 2.696 to 7.226, indicating a leptokurtic distribution (peakedness). While the data deviates from a perfect normal distribution, the sample size (n = 266) is sufficient for robust statistical analysis using methods such as Partial Least Squares Structural Equation Modeling (PLS-SEM), which handles non-normal data effectively. This dataset allows researchers to perform both parametric and non-parametric tests depending on their specific analytical requirements.

In terms of Psychological capital, the measured skew was −2.150, showing a left-skewed distribution since most competency values were in the upper or “right” part, while lower or “left” values were rare. Psychological capital had a kurtosis of 7.226, stating that the distribution of data had thicker tails and sharper peaks. Therefore, the distribution of psychological capital had significant extreme values, or there was a density of data around certain values. Regarding the employability of vocational students, the calculated skew was −1.249, signaling that the distribution was skewed to the left since most competency values were in the upper or “right” part, while lower or “left” values were rare. Vocational students’ employability reported a kurtosis of 3.345 since the distribution of data had thicker tails and sharper peaks. Therefore, this variable had significant extreme values or a density of data around certain values.

The codebook and raw dataset have been posted to the Mendeley Data Repository for public sharing [9] in Excel format (Raw Dataset (Excel).xlsx) and contains demographic information (age, gender, school status, and school accreditation), students’ internship experience (SIE1-SIE14), competence (C1-C10), psychological capital (PsyCap1-PsyCap14), and Employability of Vocational Students (EVS1-EVS12). Furthermore, a survey questionnaire written in Indonesian and English translation has been included as an additional document (survey questionnaire.docx). The data were collected and shared according to the ethical standards of Universitas Padjadjaran. Special procedures were also taken to maintain respondents’ confidentiality and data anonymity. The use must be strictly for academic and research purposes, following ethical practices.

## 4 Experimental Design, Materials, and Methods

A cross-sectional survey design was adopted to capture the employability data of vocational students. The target population comprised Grade XII vocational students across Aceh Province. According to the aggregate data from the education authorities, the total population was 15,435 students (N = 15,435). To ensure scientific rigor and representativeness, a Proportional Stratified Random Sampling technique was employed rather than simple random sampling

The minimum sample size was determined using the Isaac and Michael [1] formula with a confidence level of 95% ($\lambda^{2}$ = 2.705, df = 1) and a margin of error of 5% (d = 0.05). The proportion (P) was set at 0.5 to maximize the sample size estimation. The calculation is as follows

$$s=\;\frac{\lambda^{2}.N.P.Q}{d^{2}\left(N-1\right)+\lambda^{2}.P.Q}$$

$$s=\;\frac{2,705\;x\;15.435\;x\;0,5\;x\;0,5}{0,05^{2}\left(15.435\;-1\right)+2,705\;x\;0,5\;x\;0,5}=266$$

Based on this calculation, the required minimum sample size was 262 respondents. Our study successfully collected 266 valid responses, exceeding the minimum requirement to ensure data robustness. To ensure the sample accurately reflected the demographic distribution, respondents were selected from 17 representative vocational schools across 7 districts using proportional allocation. Table 3 details the distribution of the sample across the selected districts.

**Table 3:** Distribution of sampled schools across selected districts in Aceh province.

No	Region (Regency/City)	Population of Grade XII in District (Ni)	Final Sample Size (n)	Representative Schools Sampled
1	Langsa City	1174	42	SMKN 2 Langsa, SMK Perbankan Graha Media
2	North Aceh Regency	1546	56	SMKN 1 Dewantara, SMKN 1 Lhoksukon, SMKN 1 Muara Batu, SMKN 1 Nisam, SMKN 1 Tanah Luas
3	Lhokseumawe City	1419	51	SMKN 7, SMKN 3, SMKN 5 Lhokseumawe
4	Bireuen Regency	972	35	SMKN 1 Ganda Pura, SMKS Kes. Muhammadiyah, SMKN 1 Peusangan
5	Central Aceh Regency	673	24	SMKN 3 Takengon
6	Banda Aceh City	1399	51	SMKN 3 Banda Aceh, SMKN 5 Telkom
7	Aceh Jaya Regency	190	7	SMKN 1 Panga
**Total** **(Sampled Districts)**		**7373**	**266**	**17 Vocational Schools**

**Note**: While the total provincial population (N = 15,435) was used for sample size calculation via Isaac and Michael [1], the table above details the specific population distribution (Nselected=7373) in the 7 districts where data collection took place.

**Table 4:** Structure of the survey instrument and measurement scales.

Variable	Section	Number of Items	Measurement Dimension	Source Adoption
Students’Internship Experience	B	14	Clear objectives, School support, Working environment, Job prospects	Luk & Chan, 2020; Jawabri, 2017 [[2], [3]]
Competence	C	10	Life & career skills, Learning & innovation, IT & media skills	Trilling & Fadel, 2012 [4]
Psychological Capital	D	14	Hope, Optimism, Resilience, Self-efficacy	Luthans et al., 2011 &, 2017 [[5], [6]]
Employability of Vocational Student’s	E	12	Professional development, Career identity, Environmental monitoring, Balance	Van Der Heijde & Van Der Heijden, 2006; Lo Presti et al., 2019 [[7], [8]]

The questionnaire was administered using closed questions and translated into Bahasa Indonesia. This consisted of five sections, namely Section A: respondents and school data, Section B: students’ internship experience, Section C: psychological capital, Section D: competence, and Section EVS: employability of vocational student’s. Section A asked for the demographics of respondents and included questions on respondents’ code, gender, school status, and school accreditation. The comprehensive distribution of these sections, alongside their corresponding number of items, measurement dimensions, and original source adoptions, is systematically detailed in Table 4.

Section B consisted of fourteen items adopted from the Survey of Students’ Internship Experience [[2], [3]], and assessed four factors of the variable, namely clear internship objective, school support, comfortable working environment, and job prospects. Meanwhile, each aspect had three or four items with positively worded items. The items had a five-point Likert scale response, with 1 “strongly disagree”, 2 “disagree”, 3 “Neither Agree nor Disagree”, 4 “agree”, and 5 “strongly agree”. The sample items were as follows: “I was clear from the start about the purpose of the internship and what I learned during internship”, “The school supervisor provided support in completing the tasks and projects assigned during internship”, and “The results of my work during internship provided financial benefits or career opportunities that had a positive impact on my well-being."

Section C consisted of fourteen items adopted from the competency survey [4] and assessed four factors of the variable, namely life and career competencies, learning and innovation competencies, and information technology and media competencies. In addition, each aspect had three or four items with positively worded items. The items had a five-point Likert scale response, with 1 “strongly disagree”, 2 “disagree”, 3 “neither agree nor disagree”, 4 “agree”, and 5 “strongly agree”. The examples included: “I can adapt quickly to changing situations or demands that occur in learning or group activities,” “I can communicate clearly and collaborate with other group members,” and “I am able to select and develop media used to communicate."

Section D consisted of fourteen items adopted from the Psychological Capital survey [[5], [6]], and assessed four factors of the variable, namely hope, optimism, resilience, and self-efficacy. Furthermore, each aspect had three or four items with positively worded items. The items had a five-point Likert scale response, with 1 “strongly disagree”, 2 “disagree”, 3 “neither agree nor disagree”, 4 “agree”, and 5 “strongly agree”. The examples included: “I have the ability to find various ways to overcome obstacles that arise during my studies,” “I have a strong belief that I can realize my dreams and achieve the big goals I set,” “I have a strong belief that I can complete difficult tasks successfully."

Section E consisted of fourteen items adopted from the employability survey of vocational students [[7], [8]], and assessed four factors of the variable, namely human capital and professional development, human capital and networks, career identity and self-management, environmental monitoring, and balance. Furthermore, each aspect had three or four items with positively worded items. The items had a five-point Likert scale response, with 1 “strongly disagree”, 2 “disagree”, 3 “neither agree nor disagree”, 4 “agree”, and 5 “strongly agree”. The examples included: “I can discuss professionally with various professionals or technical aspects of work,” “I can convince others about work when team decisions have to be made,” and “I can achieve a balance between work goals and the support of colleagues.”

The items of the self-administered questionnaire survey using paper and pencil were used to collect data. The background and purpose of the article were explained to respondents. To avoid misunderstandings or incorrect answers by respondents, clear and precise instructions were provided. In this context, respondents were guided in answering questions to minimize errors. The data was extracted into Excel format and analyzed through IBM SPSS Statistics 22 for frequency, descriptive analysis, and reliability testing. Table 5 shows the Cronbach's alpha values for SIE, C, PsyCap, and EVS as 0.919, 0.907, 0.926, and 0.917, respectively.

**Table 5:** Cronbach’s alpha value of the variables.

Variable	No. of questions	Cronbach’s alpha
Students’ internship experience (SIE)	14	0919
Competence (C)	10	0907
Psychological capital (PsyCap)	14	0926
Employability vocational students (EVS)	12	0917

## Limitations

The issue of employability is often associated with students’ internships, while related discussions on the variable are limited. Since internships are not balanced with the school curriculum, this can lead to gaps in students' learning experiences, resulting in inadequate quality. In this context, optimizing students' potential and participation in the survey is difficult. According to the latest official data released by the Indonesian Ministry of Education and Culture on 30 August 2022, Aceh Province has 220 Vocational Schools with various disciplines. Due to time and contact constraints, only 17 public and private schools were considered in this article. Therefore, trials should be conducted with a larger sample of participants regarding the relationship between students' internship experience, competence, psychological capital, and employability of vocational students. Another limitation is the lack of access or opportunity for students from economically disadvantaged backgrounds or remote areas. Data were only collected on four variables, namely internship experience, competence, psychological capital, and employability. Despite the limitations, the results obtained are expected to provide new knowledge on this topic.

## Ethics Statement

This study was conducted in strict accordance with the ethical guidelines of the Declaration of Helsinki. The research protocol was approved by the Ethics Committee of Universitas Padjadjaran (Protocol Number: 79/UN6.KEP/EC/2025). Informed consent was obtained from all participants involved in the study. Before data collection, respondents were briefed on the study's objectives, and they signed a written consent form acknowledging their voluntary participation and the anonymity of their data. A blank copy of the consent form is provided as supplementary material.

## Data Availability

Dataset of students’ internship experience, competence, psychological capital, and employability (Original data) (Mendeley data) https://data.mendeley.com/datasets/hmn3b4c6c4/4.

## Declaration of Generative AI and AI-assisted Technologies in the Writing Process

During the preparation of this work the author(s) used Gemini (Google) in order to improve the readability and language quality of the manuscript. After using this tool/service, the author(s) reviewed and edited the content as needed and take(s) full responsibility for the content of the published article.

## Appendix Supplementary materials

Image, application 1

Image, application 2
