# Supplementary material for: A stratified survey dataset on internship experience, competencies, psychological capital, and employability of vocational students in Aceh, Indonesia
Source: Data Brief. 2026 Jun 20;67:112992. doi: 10.1016/j.dib.2026.112992 (PMC13333311; doi:10.1016/j.dib.2026.112992)
Supplement: Supplementary file 2 [file mmc2.docx]

**DATASET SURVEI PENGALAMAN MAGANG, KOMPETENSI, MODAL PSIKOLOGI, DAN KEMAMPUAN KERJA SISWA PADA SISWA SMK PROVINSI ACEH**

**SURVEY DATASET ON STUDENTS’ INTERNSHIP EXPERIENCE, COMPETENCIES, PSYCHOLOGICAL CAPITAL, AND EMPLOYABILITY AMONG**

**VOCATIONAL STUDENT IN ACEH PROVINCE**

**BAGIAN A. DATA RESPONDEN DAN SEKOLAH**

***Section A. Respondent and School Data***

Silahkan mengisi dengan lengkap dan mencoret yang tidak sesuai dengan data responden beserta data sekolah di bawah ini:

*Please fill in completely and cross out anything that does not match the respondent data along with the school data below:*

- Kode Responden/ *Respondent Code* : ………………………………………………………..………………….
- Jenis Kelamin/ *Gender* : Laki-Laki (*Male)* / Perempuan (*Female*)

(lingkar sesuai jawaban/ *circumference according to*

*answer*)

- Status Sekolah/ *School status :* ……………………………………………………….……………………
- Akreditasi Sekolah/ *School Accreditation :* ………………………………………………………………………………

| Tanda Tangan/ *Signature* | Stempel sekolah/ School stamp |
| --- | --- |

**BAGIAN B. PENGALAMAN MAGANG SISWA (PMS)**

***Section B. Student Internship Experience (SIE)***

Bagian quisioner ini mengandung penyataan item yang berkaitan dengan pengalaman magang siswa pada setiap aspek pekerjaan anda.

*This section of the questionnaire contains item statements relating to the student's internship experience in each aspect of your job.*

Anda cukup memilih salah satu alternatif jawaban atas penyataan item yang tersedia dengan memberi tanda ceklist ($\surd$), tanda silang (X) atau lingkaran (O) pada kotak jawaban yang tersedia.

*You simply select one of the alternative answers to the available item statements by putting a checklist (√), cross (X) or circle (O) in the answer box provided.*

| *1* | sangat tidak setuju/ *strongly Disagree* |  | *4* | Setuju/ *Agree* |
| --- | --- | --- | --- | --- |
| *2* | Tidak setuju/ *Disagree* |  | *5* | Sangat Setuju/ *Strongly agree* |
| *3* | Tidak setuju maupun tidak menolak/ *Neither Agree nor Disagree* |  |  |  |

| **No** | **Kode/ *Code*** | **PERNYATAAN ITEM/**  ***ITEM STATEMENT*** | **Skala/ *Scale*** | | | | |
| --- | --- | --- | --- | --- | --- | --- | --- |
|  |  |  | sangat tidak setuju/ *strongly* *disagree* | Tidak setuju/ *Disagree* | Tidak setuju maupun tidak menolak/ *Neither Agree nor Disagree* | Setuju/ *Agree* | Sangat Setuju/ *Strongly agree* |
| 1 | SIE1 | Sejak awal saya mendapatkan kejelasan terkait tujuan magang dan hal-hal yang dipelajari ketika magang  *From the start I got clarity regarding the purpose of the internship and the things* *I learned during the internship* | 1 | 2 | 3 | 4 | 5 |
| 2 | SIE2 | saya memperoleh pengetahuan dengan mudah dimana akan dinilai ketika magang.  *I gain knowledge easily which will be assessed during my internship* | 1 | 2 | 3 | 4 | 5 |
| 3 | SIE3 | Dengan mudah saya mengetahui standar pekerjaan ketika magang.  *I easily know the standards of work during my internship* | 1 | 2 | 3 | 4 | 5 |
| 4 | SIE4 | saya memiliki gagasan yang jelas terkait pekerjaan apa yang dilakukan selama magang  *I have a clear idea of what work to do during my internship* | 1 | 2 | 3 | 4 | 5 |
| 5 | SIE5 | sekolah sangat membantu saya dalam mempersiapkan mencari tempat magang.  *The school really helped me in preparing to find an internship* | 1 | 2 | 3 | 4 | 5 |
| 6 | SIE6 | Supervisor sekolah memberikan dukungan dalam menyelesaikan tugas-tugas dan proyek yang diberikan selama magang  *School supervisors provide support in completing assignments and projects given during the internship* | 1 | 2 | 3 | 4 | 5 |
| 7 | SIE7 | Supervisor sekolah memberikan dukungan dalam mengidentifikasi dan memahami masalah yang muncul selama magang.  *School supervisors provide support in identifying and understanding problems that arise during the internship* | 1 | 2 | 3 | 4 | 5 |
| 8 | SIE8 | Saya diperlakukan dengan hormat dan profesionalisme oleh semua pihak di tempat magang, termasuk rekan kerja dan atasan  *I was treated with respect and professionalism by all parties at the internship site, including colleagues and superiors* | 1 | 2 | 3 | 4 | 5 |
| 9 | SIE9 | Atasan magang saya selalu tersedia untuk menjawab pertanyaan dan memberikan bimbingan selama pelaksanaan magang  *My internship supervisor was always available to answer questions and provide guidance during the internship* | 1 | 2 | 3 | 4 | 5 |
| 10 | SIE10 | Supervisor industri saya memberikan pandangan alternatif dan berbagi pengalaman pribadi yang relevan dengan masalah yang saya hadapi selama magang  *My industrial supervisor provided an alternative view and shared personal experiences relevant to the problems I faced during my internship* | 1 | 2 | 3 | 4 | 5 |
| 11 | SIE11 | Supervisor industri saya secara rutin memberikan umpan balik yang jelas dan terperinci tentang kinerja saya selama magang  *My industrial supervisor regularly provided clear and detailed feedback on my performance during my internship* | 1 | 2 | 3 | 4 | 5 |
| 12 | SIE12 | Hasil pekerjaan saya selama magang memberikan manfaat finansial atau peluang karier yang berdampak positif pada kesejahteraan hidup saya.  *The results of my work during my internship provide financial benefits or career opportunities that have a positive impact on the well-being of my life*. | 1 | 2 | 3 | 4 | 5 |
| 13 | SIE13 | Saya memiliki kesempatan untuk terlibat dalam proyek-proyek yang menantang dan memberikan pengalaman berharga untuk kemajuan karier saya.  *I have the opportunity to be involved in projects that are challenging and provide valuable experience for the advancement of my career*. | 1 | 2 | 3 | 4 | 5 |
| 14 | SIE14 | Magang ini memberikan kesempatan untuk mengamati dan belajar dari praktik-praktik terbaik yang dilakukan oleh karyawan berpengalaman di Perusahaan  *This internship provides an opportunity to observe and learn from the best practices carried out by experienced employees at the company* | 1 | 2 | 3 | 4 | 5 |

**BAGIAN C. MODAL PSIKOLOGI (MP)**

***Section C. Psychological Capital (PsyCap)***

Bagian quisioner ini mengandung penyataan item yang berkaitan dengan modal psikologi siswa pada setiap aspek pembelajaran dan pekerjaan.

*This section of the questionnaire contains item statements relating to the* student's psychological capital in every aspect of learning and job

Anda cukup memilih salah satu alternatif jawaban atas penyataan item yang tersedia dengan memberi tanda ceklist ($\surd$), tanda silang (X) atau lingkaran (O) pada kotak jawaban yang tersedia.

*You simply select one of the alternative answers to the available item statements by putting a checklist (√), cross (X) or circle (O) in the answer box provided.*

| *1* | sangat tidak setuju/ *strongly Disagree* |  | *4* | Setuju/ *Agree* |
| --- | --- | --- | --- | --- |
| *2* | Tidak setuju/ *Disagree* |  | *5* | Sangat Setuju/ *Strongly agree* |
| *3* | Tidak setuju maupun tidak menolak/ *Neither Agree nor Disagree* |  |  |  |

| **No** | **Kode/ *Code*** | **Pernyataan item/**  ***Item Statement*** | **Skala/ *Scale*** | | | | |
| --- | --- | --- | --- | --- | --- | --- | --- |
|  |  |  | sangat tidak setuju/ *strongly* *disagree* | Tidak setuju/ *Disagree* | Tidak setuju maupun tidak menolak/ *Neither Agree nor Disagree* | Setuju/ *Agree* | Sangat Setuju/ *Strongly agree* |
| 1 | *PsyCap*1 | Saya memiliki kemampuan untuk menemukan berbagai cara untuk mengatasi kendala yang muncul selama studi saya  *I can find various ways to overcome obstacles that arise during my studies* | 1 | 2 | 3 | 4 | 5 |
| 2 | *PsyCap*2 | Saya menyadari bahwa ada banyak pendekatan yang bisa digunakan untuk mengatasi masalah yang dihadapi.  *I realized that many approaches can be used to overcome the problems faced*. | 1 | 2 | 3 | 4 | 5 |
| 3 | *PsyCap*3 | Saya berkomitmen untuk melakukan segala upaya terbaik dan bekerja keras untuk mencapai tujuan yang saya tetapkan  *I am committed to doing my best and working hard to achieve the goals I set* | 1 | 2 | 3 | 4 | 5 |
| 4 | *PsyCap*4 | Saya tetap memiliki harapan yang tinggi bahwa dapat berhasil dalam situasi pembelajaran di masa pandemi.  *I still have high hopes that I can succeed in learning situations during the pandemic*. | 1 | 2 | 3 | 4 | 5 |
| 5 | *PsyCap*5 | Saya optimis bahwa investasi waktu dan usaha yang saya lakukan saat ini akan membawa hasil yang positif di masa depan.  *I am optimistic that the investment of time and effort I am making now will bring positive results in the future*. | 1 | 2 | 3 | 4 | 5 |
| 6 | *PsyCap*6 | Saya cenderung melihat pelajaran dan pembelajaran yang positif dari setiap pengalaman belajar, baik sukses maupun kegagalan.  *I tend to see positive lessons and learnings from every learning experience, both success and failure*. | 1 | 2 | 3 | 4 | 5 |
| 7 | *PsyCap*7 | Saya memiliki keyakinan yang kuat bahwa saya dapat mewujudkan mimpi dan mencapai tujuan besar yang saya tetapkan.  *I have a strong belief that I can make my dreams come true and achieve the big goals I set*. | 1 | 2 | 3 | 4 | 5 |
| 8 | *PsyCap*8 | Saya memiliki kemampuan untuk mengidentifikasi dan menganalisis berbagai masalah yang muncul dalam pembelajaran  *I can identify and analyze various problems that arise in learning* | 1 | 2 | 3 | 4 | 5 |
| 9 | *PsyCap*9 | Saya selalu berusaha mengetahui penyebab dari nilai rendah yang saya terima dan mencari solusi untuk memperbaikinya  *I always try to find out the cause of the low grades I receive and find solutions to improve them* | 1 | 2 | 3 | 4 | 5 |
| 10 | *PsyCap*10 | Saya memahami bahwa setiap orang mengalami kegagalan dan bangkit kembali adalah kunci untuk mencapai kesuksesan jangka Panjang  *I understand that everyone experiences failure and getting back up is the key to achieving long-term success* | 1 | 2 | 3 | 4 | 5 |
| 11 | *PsyCap*11 | Saya memiliki motivasi intrinsik yang kuat untuk mencapai prestasi akademik yang tinggi dan selalu mencari cara untuk meningkatkan diri secara terus-menerus.  *I have a strong intrinsic motivation to achieve high academic achievements and am always looking for ways to continuously improve myself* | 1 | 2 | 3 | 4 | 5 |
| 12 | *PsyCap*12 | Saya memiliki keyakinan yang kuat bahwa saya dapat menyelesaikan tugas yang sulit dengan sukses.  *I have a strong belief that I can complete difficult tasks successfully* | 1 | 2 | 3 | 4 | 5 |
| 13 | *PsyCap*13 | Saya memiliki keyakinan diri yang cukup untuk bertanya kepada guru ketika mengalami kesulitan dalam mengerjakan tugas  *I have enough self-confidence to ask the teacher when I have difficulty doing an assignment* | 1 | 2 | 3 | 4 | 5 |
| 14 | *PsyCap*14 | Saya merasa percaya diri bahwa saya dapat memberikan kontribusi yang berarti dan bermanfaat kepada orang lain dengan kemampuan yang saya miliki.  *I feel confident that I can make a meaningful and useful contribution to others with the abilities I have* | 1 | 2 | 3 | 4 | 5 |

**BAGIAN D. KOMPETENSI (K)**

***Section D. Competence (C)***

Bagian quisioner ini mengandung penyataan item yang berkaitan dengan kompetensi siswa pada setiap aspek pembelajaran dan pekerjaan.

*This section of the questionnaire contains item statements relating to the* students' competence in every aspect of learning and job

Anda cukup memilih salah satu alternatif jawaban atas penyataan item yang tersedia dengan memberi tanda ceklist ($\surd$), tanda silang (X) atau lingkaran (O) pada kotak jawaban yang tersedia.

*You simply select one of the alternative answers to the available item statements by putting a checklist (√), cross (X) or circle (O) in the answer box provided.*

| *1* | sangat tidak setuju/ *strongly Disagree* |  | *4* | Setuju/ *Agree* |
| --- | --- | --- | --- | --- |
| *2* | Tidak setuju/ *Disagree* |  | *5* | Sangat Setuju/ *Strongly agree* |
| *3* | Tidak setuju maupun tidak menolak/ *Neither Agree nor Disagree* |  |  |  |

| **No** | **Kode/ *Code*** | **PERNYATAAN ITEM/**  ***ITEM STATEMENT*** | **Skala/ *Scale*** | | | | |
| --- | --- | --- | --- | --- | --- | --- | --- |
|  |  |  | sangat tidak setuju/ *strongly* *disagree* | Tidak setuju/ *Disagree* | Tidak setuju maupun tidak menolak/ *Neither Agree nor Disagree* | Setuju/ *Agree* | Sangat Setuju/ *Strongly agree* |
| 1 | C1 | Saya memiliki kemampuan untuk beradaptasi dengan cepat terhadap perubahan situasi atau tuntutan yang terjadi dalam pembelajaran atau kegiatan kelompok.  *I have the ability to adapt quickly to changing situations or demands that occur in learning or group activities*. | 1 | 2 | 3 | 4 | 5 |
| 2 | C2 | Saya memiliki kemampuan untuk berinteraksi dengan jelas dan efektif dengan anggota kelompok yang memiliki latar belakang, kepercayaan, atau pendapat yang berbeda-beda.  *I have the ability to interact clearly and effectively with group members who have different backgrounds, beliefs or opinions*. | 1 | 2 | 3 | 4 | 5 |
| 3 | C3 | Saya memiliki kemampuan untuk merencanakan, mengorganisir, dan menjalankan proyek dengan efisien dan efektif.  *I have the ability to plan, organise and execute projects efficiently and effectively*. | 1 | 2 | 3 | 4 | 5 |
| 4 | C4 | Saya memiliki kemampuan untuk memimpin dan menginspirasi anggota kelompok dengan visi, tujuan, dan nilai-nilai yang jelas.  *I have the ability to lead and inspire group members with a clear vision, purpose and values*. | 1 | 2 | 3 | 4 | 5 |
| 5 | C5 | saya mampu menggunakan berbagai alasan secara induktif atau deduktif untuk berbagai situasi; menggunaan cara berpikir system dan membuat keputusan.  *I am able to use inductive or deductive reasoning for a variety of situations; use systems thinking and decision making*. | 1 | 2 | 3 | 4 | 5 |
| 6 | C6 | saya mampu berkomunikasi dengan jelas dan melakukan kolaborasi dengan anggota kelompok lainnya.  *I was able to communicate clearly and collaborate with other group members* | 1 | 2 | 3 | 4 | 5 |
| 7 | C7 | saya mampu berpikir, bekerja secara kreatif, serta menciptakan inovasi baru.  *I am able to think work creatively and create new innovations*. | 1 | 2 | 3 | 4 | 5 |
| 8 | C8 | Saya mampu mengakses informasi secara efektif dan efesien serta mengevaluasi informasi yang digunakan secara kompeten, dan efektif untuk megatasi masalah.  *I am able to access information effectively and efficiently and evaluate the information used competently, and effectively to solve problems*. | 1 | 2 | 3 | 4 | 5 |
| 9 | C9 | Saya mampu memilih dan mengembangkan media yang digunakan untuk berkomunikasi.  *I am able to select and develop media used for communication* | 1 | 2 | 3 | 4 | 5 |
| 10 | C10 | saya mampu menganalisis media informasi dan menciptakan media yang sesuai untuk melakukan komunikasi.  *I was able to analyse information media and create appropriate media for communication*. | 1 | 2 | 3 | 4 | 5 |

**BAGIAN E. EMPLOYABILITY SISWA KEJURUAN (ESK)**

***SECTION E. Employability of Vocational Student’s (EVS)***

Bagian quisioner ini mengandung penyataan item yang berkaitan dengan employability siswa kejuruan pada setiap aspek pembelajaran dan pekerjaan.

*This section of the questionnaire contains item statements relating to vocational student’s employability in every aspect of learning and job.*

Anda cukup memilih salah satu alternatif jawaban atas penyataan item yang tersedia dengan memberi tanda ceklist ($\surd$), tanda silang (X) atau lingkaran (O) pada kotak jawaban yang tersedia.

*You simply select one of the alternative answers to the available item statements by putting a checklist (√), cross (X) or circle (O) in the answer box provided.*

| *1* | sangat tidak setuju/ *strongly Disagree* |  | *4* | Setuju/ *Agree* |
| --- | --- | --- | --- | --- |
| *2* | Tidak setuju/ *Disagree* |  | *5* | Sangat Setuju/ *Strongly agree* |
| *3* | Tidak setuju maupun tidak menolak/ *Neither Agree nor Disagree* |  |  |  |

| **No** | **Kode/ *Code*** | **Pernyataan Item/**  ***Item Statement*** | **Skala/ *Scale*** | | | | |
| --- | --- | --- | --- | --- | --- | --- | --- |
|  |  |  | sangat tidak setuju/ *strongly* *disagree* | Tidak setuju/ *Disagree* | Tidak setuju maupun tidak menolak/ *Neither Agree nor Disagree* | Setuju/ *Agree* | Sangat Setuju/ *Strongly agree* |
| 1. | EVS1 | saya memiliki kemampuan dalam diskusi secara professional dengan berbagai profesi atau aspek teknis pekerjaan.  *I have the ability to discuss professionally with various professionals or technical aspects of work* | 1 | 2 | 3 | 4 | 5 |
| 2. | EVS2 | saya mencapai tingkat pendidikan yang memadai dalam beroperasi sebagai calon karyawan baru dalam pekerjaan.  *I achieved a level of education sufficient to operate as a prospective new employee in the job*. | 1 | 2 | 3 | 4 | 5 |
| 3. | EVS3 | saya mampu untuk mengevaluasi keterampilan teknis yang memadai dapat menyelesaikan tugas atau memecahkan masalah di sekolah.  *I am able to evaluate sufficient technical skills to be able to complete tasks or solve problems at school* | 1 | 2 | 3 | 4 | 5 |
| 4. | EVS4 | saya memiliki tingkat pengalaman profesional yang cukup memuaskan untuk beroperasi dalam pekerjaan.  *I have a satisfactory level of professional experience to operate on the job*. | 1 | 2 | 3 | 4 | 5 |
| 5. | EVS5 | saya mampu meyakinkan orang lain tentang pekerjaan, ketika keputusan dalam tim harus dibuat.  *I am able to convince others about work when decisions within the team have to be made* | 1 | 2 | 3 | 4 | 5 |
| 6. | EVS6 | membangun hubungan sosial dapat memiliki efek positif pada hidup saya  *Building social relationships can have a positive effect on my life* | 1 | 2 | 3 | 4 | 5 |
| 7. | EVS7 | saya memiliki rencana yang jelas untuk karir  *I have a clear plan for my career* | 1 | 2 | 3 | 4 | 5 |
| 8. | EVS8 | saya memilki tujuan karir yang jelas  *I have clear career goals* | 1 | 2 | 3 | 4 | 5 |
| 9. | EVS9 | saya memiliki strategi yang jelas untuk mencapai tujuan karir  *I have a clear strategy to achieve my career goals* | 1 | 2 | 3 | 4 | 5 |
| 10. | EVS10 | saya memiliki gagasan yang jelas tentang target industri sesuai dengan bidang pekerjaan.  *I have a clear idea of the target industry according to my field of work.* | 1 | 2 | 3 | 4 | 5 |
| 11. | EVS11 | saya mampu mencapai keseimbangan antarai tujuan kerja dengan dukungan rekan kerja  *I am able to achieve a balance between my work goals and the support of my colleagues*. | 1 | 2 | 3 | 4 | 5 |
| 12. | EVS12 | saya mampu mencapai keseimbangan antara tujuan karir dengan dukungan rekan kerja.  *I am able to achieve a balance between my career goals and the support of my colleagues*. | 1 | 2 | 3 | 4 | 5 |

**Penjelasan tambahan terkait hal di atas bila diperlukan**

*Additional explanations related to the above if necessary*

| ***Kesan yang tidak sesuai harapan selama magang***  *Impressions that did not match expectations during the internship* |
| --- |

** $\sim\sim$ ***TERIMA KASIH ATAS PARTISIPASINYA*** $\sim\sim$ **

** $\sim\sim$ *THANK YOU FOR YOUR PARTICIPATION* $\sim\sim$ **
